# Dynamic Features of Herd Immunity: Similarities in Age-Specific Anti-Measles Seroprevalence Data between Two Countries of Different Epidemiological History

**DOI:** 10.3390/jcm11041145

**Published:** 2022-02-21

**Authors:** Katalin Böröcz, Senka Samardžić, Ines Drenjančević, Ákos Markovics, Tímea Berki, Péter Németh

**Affiliations:** 1 Department of Immunology and Biotechnology, Medical School, University of Pécs, 7624 Pécs, Hungary; berki.timea@pte.hu (T.B.); nemeth.peter@pte.hu (P.N.); 2 Department of Public Health, Teaching Institute of Public Health for The Osijek-Baranja County, 31000 Osijek, Croatia; senka.007@gmail.com; 3 Institute and Department of Physiology and Immunology, Faculty of Medicine Osijek, Josip Juraj Strossmayer University of Osijek, 31000 Osijek, Croatia; ines.drenjancevic@mefos.hr; 4 Scientific Centre for Excellence for Personalized Health Care, Josip Juraj Strossmayer University of Osijek, 31000 Osijek, Croatia; 5 Department of General and Physical Chemistry, Faculty of Natural Sciences, University of Pécs, 7624 Pécs, Hungary; markovics424@gmail.com

**Keywords:** measles, MMR, seropositivity, susceptibility, Croatia, Hungary, vaccination, humoral, protection, dynamics

## Abstract

(1) Background: Measles immunization gap(s) raise the concern of potential outbreaks. Both Croatia and Hungary are situated in the vicinity of measles-endemic countries. Potentially compromised immunization activities due to the COVID-19 surge is a ground for concern. Our aim was to compare age-stratified seroprevalence results in the cross-border region. (2) Methods: Anti-MMR specific antibody levels (IgG) of 950 anonymous Croatian samples were compared with previous Hungarian results (*n* > 3500 samples), and former Croatian seroprevalence data (*n* = 1205). Seropositivity ratios were determined using our self-developed anti-MMR indirect ELISA (Euroimmun IgG ELISA kits were used as control). (3) Results: Measured seropositivity ratios of the Croatian samples were largely overlapping with our earlier published Hungarian data (the lowest seropositivity ratios were measured among individuals of 34–43 years of age with 78% of seropositivity) and are in accordance with earlier published data of Croatian researchers. (4) Conclusion: Although the epidemiological histories of the two countries are different, analogies in age-specific measles susceptibility have been discovered. We suggest that besides the potential coincidence in vaccination ineffectiveness, the inherent biological dynamics of vaccination-based humoral protection might have also contributed to the experienced similarities. Our findings may also serve as a lesson regarding the current anti-COVID-19 vaccination strategy.

## 1. Introduction

In Hungary, the MMR vaccine has been mandatory since 1969, with the current vaccine coverage estimated at 99% (WHO). Despite it, latent susceptible age-specific cohorts among the domestic population might be present [1,2,3,4,5,6]. Small-scale outbreaks (2017—the outbreak linked to the region of Makó and Szeged [7]) confirm that certain measles vaccines—applied during the early phases of the Hungarian vaccination history—failed to elicit the desired immunological response. The resulting immunization gap(s) raise the concern of potential further outbreaks [3,8]. Taking in consideration the risk of being in the geographical vicinity of measles-endemic country(ies), aggravated by the hazard of suspended immunization activities due to the COVID-19 surge (according to a recent WHO joint report, in 2020 more than 22 million infants missed their first dose of measles vaccine, three million more than in 2019 [9]) the importance of constant sero-epidemiological screening—also at international level—is indisputable.

Measles vaccination was introduced in Croatia in 1968. Since its introduction, Croatia has never experienced a measles (or MMR) vaccine shortage. In the last 50 years, vaccination coverage has been sufficiently high to ensure population-level immunity and to achieve the interruption of indigenous measles virus (MV) circulation [10]. Only individual, imported measles cases have occurred in the last two decades, with very limited transmission rates [10]. As with Hungary, MV outbreaks in 2018–19 in Dubrovnik, Split, Slavonski Brod, and Zagreb demonstrated that vaccination coverage may be suboptimal in certain cluster(s) of the population [10]. Additionally, from 2011 to 2017, childhood vaccination rates in Croatia showed a declining trend, which may become a precursor to a measles resurgence [10]. 

Our aim was to show the preliminary results of an age-stratified serologic survey that provide insight into the current, presumable gaps of measles-specific immunity in Croatia, compared with Hungary. By comparing the estimates of age-specific seroprevalence data, we were interested if similarities are detectable between the two countries. 

## 2. Materials and Methods

Seroprevalence study was conducted using 950 anonymous Croatian samples in comparison with previous (also anonymous) Hungarian results [7,8,11], and previous findings from Croatian researchers [12,13]. (Ethical License number 5726/2015, 8216/2020, 381-19-18/2019) Serum specimens from Croatia were stored at −80 °C and transported frozen to the Department of Immunology and Biotechnology (University of Pécs, Hungary). Measles-specific IgG antibodies were determined using our self-developed (previously published) anti-MMR indirect ELISA assay [7,8,11]. Coating antigens: Bio-Rad PIP013 measles virus, Edmonston strain, 2.8 µg/mL / Bio-Rad PIP014 Mumps virus, Enders strain, 3 µg/mL/Bio-Rad PIP044 Rubella virus, HPV-77 strain, 0.4 µg/mL. Standards and quality control reagent used for the standard curve: 3rd WHO International Standard for Anti-Measles (NIBSC code: 97/648)/Anti-Mumps Quality Control Reagent Sample 1 (NIBSC code: 15/B664)/Anti-Rubella Immunoglobulin 1st WHO International Standard Human (NIBSC code: RUBI-1-94). Color detection: polyclonal anti-human IgG HRP-conjugated (Dako polyclonal rabbit anti-human IgG or equivalent) + TMB. Pre-coated high-binding polystyrene plates (antigens were dissolved in Bio-Rad BUF030, overnight, 4–6 °C) were blocked with our self-developed, synthetic blocking buffer. Samples were applied after IgM reduction pre-treatment (Bio-Rad BUF038), to achieve an optimal signal-to-noise ratio with maximal matrix equalization. Anti-measles, mumps and rubella antibody measurements were executed simultaneously, using the automated Siemens BEP 2000 Advance system. Uniform incubation times (3 × 17 min, 37 °C) and 5-times washing circles were used. Color reaction was detected at λ = 450–620 nm. For result quantification 4-parametric logistic fit was applied. Reference ranges were set as <0.15 mIU/mL, <0.15 arbitrary U/mL and <9.5 mIU/mL for measles, mumps and rubella, respectively. As a control tests, Euroimmun (EUROIMMUN Medizinische Labordiagnostika AG, Lübeck, Germany) anti-measles, mumps and rubella virus IgG ELISAs were used. Qualitative seroprevalence results (positive, negative, equivocal) of anti-measles-, mumps and rubella specific antibodies were stratified simply by ‘age decades’, since discrepancies were found in the literature data regarding historical vaccination schedules. (Important milestones of the Croatian and Hungarian measles/MMR vaccination history are summarized in Supplementary Tables S1 and S2, Tissue Culture Infectious Dose (TCID_50_) of the measles, mumps, and rubella components of different MMR and MMRV vaccines are summarized in Supplementary Table S3).

Residual sera obtained during routine laboratory sampling were collected. Samples derive from a variety of geographical locations within Croatia (cities of Zagreb, Rijeka, Varaždin, Osijek and Slavonski Brod) to provide a reasonably representative estimate of the general population immunity. The only specimen-related data used for this survey was the date of birth. Sera were collected regardless of vaccination status, immune status, or history of measles. 

## 3. Results

### 3.1. Comparison of the Croatian Seropositivity Ratios to Hungarian Data

**Figure 1:**
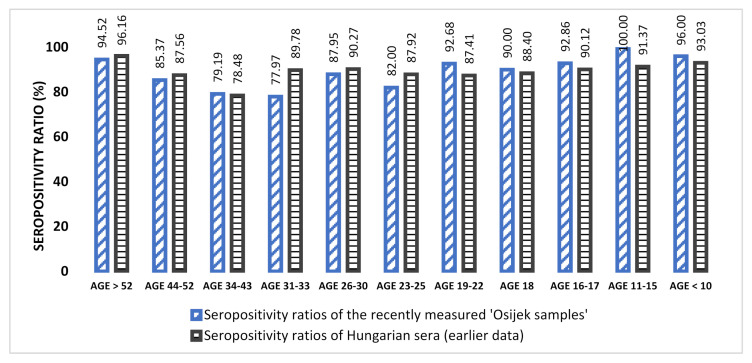
Comparison of the Croatian seropositivity ratios to Hungarian data. For the comparison with our previous sero-epidemiological results (*n* = 3919) we stratified the recently measured Croatian samples according to the already established age clusters of the Hungarian samples (*n* = 924). (Further details on the compared sample numbers can be found in Supplementary Table S4).

Interestingly, the recently measured and herein-presented seropositivity ratios (Figure 1, Supplementary Table S4) of the Croatian samples are in accordance with earlier published Hungarian data: the lowest seropositivity ratios were measured among individuals of 31–43 years of age. 

**Figure 2:**
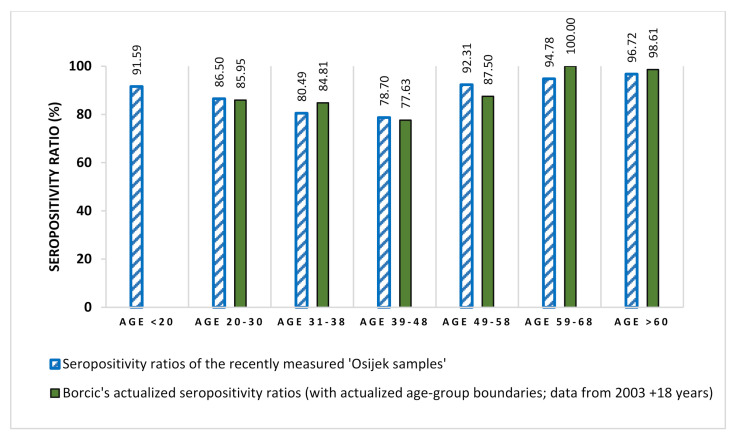
Comparison of the recently measured Croatian seropositivity ratios with previous Croatian data. For the comparison with the earlier published Croatian protection rates (Borcic et al., 2003, *n* = 1205), we distributed the samples into age groups defined by Borcic and colleagues. Our current results (*n* = 941) resemble to the actualized results of earlier literature data. (Further details on the compared sample numbers can be found in Supplementary Table S5).

By actualizing previously published Croatian protection rates (data from Borcic et al., 2003 + 18 years), we discovered that our current results are in accordance with earlier findings (Figure 2, Supplementary Table S5). This is also a useful verification regarding the reproducibility of serological data.

### 3.2. Hypothetical Timeline of the Dynamics of Sero-Epidemiological Protection Levels, through the Example of Measles as Vaccine-Preventable Disease

Although in the two countries of our comparison (Hungary and Croatia) different vaccination schedules had been used, the potentially susceptible clusters are largely overlapping. In connection with this phenomenon—and based on our large-scale sero-epidemiological measurements, we established a hypothetical timeline that depicts the temporal evolution of immunological protection, in the case of vaccine-preventable diseases (Figure 2).

## 4. Discussion

Although in the two countries of our comparison (Hungary and Croatia) different vaccination schedules had been used, the potentially susceptible clusters are largely overlapping. 

Regarding Hungary, the explanation of protection gaps is straightforward, as has been already described [1,3,7,8]: suboptimal seropositivity ratios of determinate age clusters are likely the consequence of a combination of adverse factors. Primary vaccine failure, poorly defined (premature) age at vaccination, and potential inconsiderate handling of the thermo-instable inoculum all might have compromised vaccination efficacy [1,4]. Regarding Croatia, we have much less evidence, especially in connection with the times blighted by harsh military conflicts and the subsequent migration of war refugees. It must also be mentioned that in many countries, the previously used tissue culture infectious doses (TCID_50_) have been reduced after the termination of wild-type virus circulation (e.g., from TCID_50_ measles/mumps/rubella 10^4^/3 × 10^3^/3 × 10^3^ to 3 × 10^3^/1 × 10^3^/1 × 10^3^). (TCID_50_ of the measles, mumps, and rubella components of different MMR and MMRV vaccines are summarized in Supplementary Table S3).

**Figure 3:**
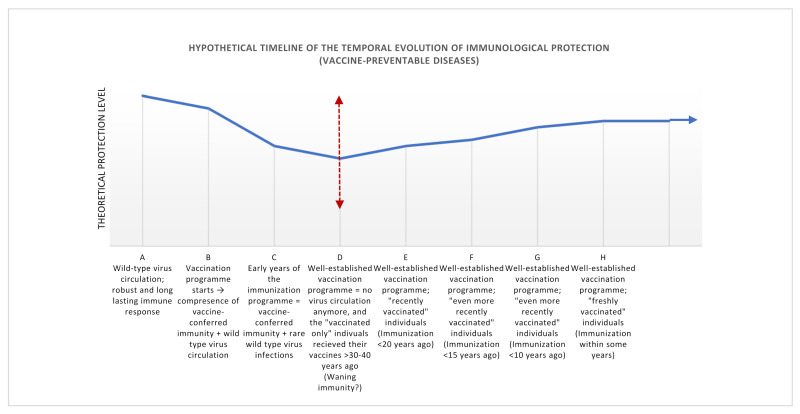
Hypothetical timeline of the dynamics of sero-epidemiological protection levels, through the example of measles. (**A**) Wild-type virus circulation followed by robust immune response and life-long present antiviral antibodies. (**B**) Vaccination program starts; contemporaneous presence of vaccine-conferred immunity and wild-type virus circulation characterize this period. (**C**) Initiatory phase of the vaccination program; vaccine-conferred immunity and remarkably decreased incidence of wild-type virus infections are simultaneously present. (**D**) Well-established vaccination program; termination of wild-type virus circulation. At the same time, post-vaccination times of the early vaccinees (>30–40 years after immunization) might be of concern, due to the phenomenon of waning immunity. (**E**) Well-established, long-ongoing vaccination program and shortened post-vaccination times (<20 years) characterize this cluster. (**F**,**G**) The continual and successful immunization program accompanied by even more recently vaccinated individuals cause the recovery of humoral protection levels in the younger age groups. (**H**) At an optimal vaccination coverage, freshly vaccinated (and boostered) young individuals show ideal seroconversion rates yielding a ‘close-to-perfect’ protection level.

Nevertheless, it is noticeable that between two geographical areas of different immunization history, the trend of anti-measles protection levels looks quite similar. Moreover, in a recent publication from Italy, Anichini et al. describe a similar trend in the age-specific IgG prevalence of the examined samples [14]. The herein-listed analogies between countries of dissimilar measles vaccination histories support the theory that besides the possibility of coincident epidemiological episodes, the natural dynamics of the transition from wild-type virus infection-induced to vaccine-delivered immunological protection might also be responsible (Figure 3). 

We suppose that a noticeable decline of seropositivity ratios at a certain time point may also be part of the intrinsic biological feature of vaccination dynamics. Today we know that immune response to wild-type measles virus infection is more robust and also more durable than that conferred by the live attenuated virus of the vaccine [15,16,17,18]. Antibody titers from samples belonging to the era of wild-type virus circulation show the highest antibody titers, and due to the high infectivity of the virus, also the higher seropositivity ratios (Figure 3). Although unvaccinated individuals account for most cases in recent measles outbreaks, the role of immune waning remains unclear [19]. As the proportion of population immunity via vaccination gradually increases and boosting through natural exposures becomes rare, risk of outbreaks may increase [19]. 

However, it can be deceptive that at a certain point immunological protection seems to be weakened, despite the already established and proven vaccination protocols. We hypothesize that this prominent decrease of the humoral response could be not necessarily due to vaccine failure, but to the synergy of two important factors: (i) measles-specific antibody titers after vaccination are lower than after natural infection, and (ii) in the case of samples with extended post-vaccination times, the phenomenon of waning immunity can be observed [14]. In summary, at the time point when the wild-type virus does not infect anymore, thus the immunological protection is based solely on the vaccine, and contemporaneously, post-vaccination become prolonged, a natural decline in humoral protection levels can be expected. Vaccinees belonging to this time window might be endangered in the case of an unexpected epidemiological episode. Therefore, it is important to delineate the potentially affected individuals country by country, to avoid the expansion of age-specific susceptibility.

Moreover, the concept of herd immunity seems to be a much more complicated concept than we previously thought. There are several factors in connection with herd immunity that despite always being present, have been pronounced only recently. 

Fine et al. [20] described some theoretical developments in the concept of herd immunity, and named an important group that is of high priority today, in relation to the COVID-19 pandemic: ‘freeloaders’ [21]. It describes people who wish that everyone else around them is vaccinated except themselves. They take the advantage of herd immunity without taking the trouble of receiving the vaccine. If immunity wanes over time, as in the case of pertussis or measles, there is a risk of focal outbreaks around the freeloaders [20,21]. Harunor et al. call attention to a key feature that is often not calculated at the evaluation of biological experiments and herd immunity threshold estimations; however, it is a unique and key attribute of humans: behavior [21]. As we experience today, living in the middle of the COVID-19 pandemic, this is among the main factors that determine the success of a vaccination strategy.

## 5. Conclusions

We believe that the herein-detailed phenomenon regarding the vulnerability of vaccine-induced humoral protection—that lies in its inherent biological dynamics—is important to address, because it may eventuate a false sense of immunological protection. Vaccinees belonging to the concerned time window might be at risk, even though they had been properly vaccinated. Hence, the importance of screening of the potentially affected age clusters is evident.

On the other hand, herd immunity seems to be a much more complicated argument than we have previously thought. These days it has become evident that besides its obvious biological complexity, human behavior—also significantly influenced by social media—must be taken in consideration as one of the main determining factors.

With the global healthcare system already proven by the COVID-19 pandemic, measles transmission—even at small scale—should be avoided. Furthermore, in the current critical epidemiological situation an adequately quick measles diagnosis may be compromised by the highly variable symptoms of the constantly mutating SARS-CoV-2 virus that are the absolute focus of medical attention. Consequently, in the unfavorable case of tardy diagnosis, large-scale spreading of measles—which has one of the highest basic reproduction numbers (R0 = 12–18) among the currently known human viruses—can be expected. Therefore, the maintenance of high immunization coverage is essential, and the existence of sufficient immunization coverage must be strictly monitored. Moreover, since the onset of the COVID-19 pandemic, the already suboptimal measles surveillance has worsened [22]. According to a recent CDC MMWR report, no WHO region has achieved and maintained measles elimination [22]. Therefore, we must maintain the suspicion for measles among international travelers with symptoms of febrile rash. Measles outbreaks of recent years should remind us to stay vigilant with the epidemiology of highly transmissible diseases, in addition to COVID-19 [23].

## 6. Limitations

The accuracy of the parallelism between Croatian and Hungarian vaccination protocol-related seroprevalence can be enhanced by contrasting the relevant historical immunization schedules of the two countries. Besides the ambivalence found in Croatian literature data [12,13], an exact ‘vaccination group-to-vaccination group’-type of comparison was encumbered by lack of sufficiently detailed Croatian epidemiological data, and differences between the two countries measles (and later MMR) vaccination schedules. For this reason, we used the literature-based comparison methods (described in the ‘Materials and Methods’ section). We summarized our current knowledge of the vaccination histories of the two countries in Supplementary Table S1 and Table S2. Our future research goal is to refine vaccination history-related data, and evaluate an extended and measured dataset (of an enlarged sample multitude) accordingly.

## Data Availability

Data are available using the following link (Google Drive, public file sharing): https://drive.google.com/drive/folders/1Z-hJYhwswbgu0xTxbqtrabo3vqTj_LcR?usp=sharing (accessed on 4 February 2022). Upon request (borocz.katalin@pte.hu) we readily provide additional data.

## Supplementary Materials

The following are available online at www.mdpi.com/article/10.3390/jcm11041145/s1, Table S1: Croatian measles /MMR vaccination history, Table S2: Hungarian measles /MMR vaccination history, Table S3: Tissue Culture Infectious Dose (TCID_50_) of the measles, mumps, and rubella components of different MMR and MMRV vaccines, Table S4: Detailed sample numbers for the comparison represented in Figure 1, Table S5: Detailed sample numbers for the comparison represented in Figure 2. Refs. [24,25,26,27,28] are cited in the Supplementary Materials.
