# Supplementary material for: Dynamic Features of Herd Immunity: Similarities in Age-Specific Anti-Measles Seroprevalence Data between Two Countries of Different Epidemiological History
Source: J Clin Med. 2022 Feb 21;11(4):1145. doi: 10.3390/jcm11041145 (PMC8879765; doi:10.3390/jcm11041145)
Supplement: Supplementary file 1 [file jcm-11-01145-s001.zip › jcm-1605980-supplementary materials.pdf]

Supplementary Materials

Table S1. Croatian measles /MMR vaccination history.

Year	Vaccine	Additional Information
1963- 1974	Monovalent (from 1973: measles-rubella bivalent) measles vaccine (Authentic data from the Croatian partner)	Immunization against measles was introduced into the official immunization schedule in Croatia in 1968 as a primary vaccination at the age of 11 months and in the first grade of elementary school. At the same time, some earlier cohorts of children in kindergartens and nursery schools were also vaccinated, so as to cover quickly the whole segment of the pre-school population [12]
1968		Immunization against measles was introduced into the official immunization schedule in Croatia in 1968 as a primary vaccination at the age of 11 months and in the first grade of elementary school [12]
1973	measles- rubella bivalent vaccine	In 1973 the primary vaccination shifts to children 1-year old and rubella is added [12]
1974	MMR vaccine	In 1974 the mumps component is added [12]
1975 -2007	MoPaRu	Who was until born 1974, received only the monovalent Measles vaccine. Who was born from 1975 to 2007 received the 'MoPaRu' (vaccine against measles, rubella and parotitis, live, lyophilized, Edmonston-Zagreb, HDS; RA 27/3, HDS; L-Zagreb, PF, Institute of Immunology, Zagreb)
1976-1994		In 1976: trivalent MMR vaccine for the 1stdose. Since 1994 trivalent vaccine was used also for the second dose [13]
1994-2009		Due to adverse events caused by the mumps component of MMR, this vaccine was replaced for the 1st dose by one of another producer in 2009 [13]
2008-2009	Priorix (GSK)	Priorix: measles, rubella and parotitis vaccine, live, lyophilized, Schwarz, PF; Wistar RA 27/3, HDS; RIT 4385, PF, GlaxoSmithKline; measles, rubella and patotitis vaccine, live, lyophilized, Edmonston-Zagreb, HDS; RA 27/3, HDS; L-Zagreb, PF, Institute of Immunology, Zagreb (Authentic data from the Croatian partner)
2010	Priorix (GSK), M-M-RVAXPro (Merck Sharp & Dohme) + Inst.of Imm.-Zagreb	Priorix: Vaccine against measles, rubella and parotitis, live, lyophilized, Schwarz, PF; Wistar RA 27/3, HDS; RIT 4385, PF, GlaxoSmithKline; MMR: live, lyophilized, Edmonston-Zagreb, HDS; RA 27/3, HDS; L-Zagreb, PF, Institute of Immunology, Zagreb; M-M-RVAXPro: Enders' Edmonston strains, Jeryl Lynn, Wistar RA 27/3, MSD (Authentic data from the Croatian partner)

2011 -2014	Priorix (GSK), M-M-RVAXPro (Merck Sharp & Dohme)	Priorix: Vaccine against measles, rubella and patotitis, live, lyophilized, Schwarz, PF; Wistar RA 27/3, HDS; RIT 4385, PF, GlaxoSmithKline; M-M-RVAXPro: strains Enders' Edmonston, Jeryl Lynn, Wistar RA 27/3, MSD.(Authentic data from the Croatian partner)
2015 - …	Priorix (GSK)	Priorix: Vaccine against measles, rubella and patotitis, live, lyophilized, Schwarz, PF; Wistar RA 27/3, HDS; RIT 4385, PF, GlaxoSmithKline (Authentic data from the Croatian partner)
According to the current protocol, children in Croatia get vaccinated age 12 months, and in the first school grade. Both vaccines are mandatory.		

Notes: Data were gathered using timely literature [12,13] and personal communication from the Croatian co-authors. Data refinement and detailing would be necessary to enable a ‘vaccination schedule-to- vaccination schedule’ or ‘vaccination group-to- vaccination group’ type of comparison between the two countries.

Table S2. Hungarian measles /MMR vaccination history.

Age-groups	Explanation, rationale
Patients born before 1969	Unvaccinated patients, wild-type infections. 1969: introduction of measles vaccine in Hungary (live, attenuated Leningrad-16 strain produced in the Soviet Union).
Patients vaccinated between 1969 - 1977	From 1969 to 1974, a single dose of measles vaccine was administered in mass campaigns to persons 9-27 months of age. The recommended age for vaccination was 10 months until 1978, when it was changed to 14 months. After the 1980-81 epidemics, persons born between 1973 and 1977, who would have received vaccine when the recommended age was 10 months, were revaccinated. After 1989, children were re-vaccinated at the age of 11 years with monovalent measles vaccine in a scheduled manner. Consequently, the first individuals who received a reminder vaccine at the age of 11 were born in 1978. Thus, the cluster of 1969-77 was the last that did not receive a reminder vaccine at the age of 11 as a part of the official vaccine schedule [4].
Patients vaccinated between 1978 - 1987	These are the first individuals who benefited from the reminder monovalent measles vaccine at the age of 11. In 1999 the administration of trivalent vaccine was started in Hungary, consequently who received the first trivalent vaccine in 1999 were born in 1988 [4].
Patients vaccinated between 1988 - 1990	In 1989 the rubella vaccine was introduced, and the monovalent measles reminder vaccine at age 11 was started.<br>1990: Introduction of measles-rubella bivalent vaccines [24].
Patients vaccinated between 1991 - 1995	The administration of the first vaccine at age 14 months lasted from 1978 to 1991.<br>1991: Measles-mumps-rubella trivalent vaccine<br>1992: MMR vaccine at age 15 months<br>1996: Introduction of MERCK MMR II - Enders' Edmonston strain (live, attenuated)
Patients born between 1996 – 1998	1996: Introduction of MERCK MMR II - Enders' Edmonston strain (live, attenuated)<br>1999: Measles-mumps-rubella re-vaccination (reminder shot) instead of monovalent measles vaccine<br>1999: Introduction of GSK PLUSERIX - Measles Schwarz Strain
Patients vaccinated between 1999 - 2002	1999: Introduction of GSK PLUSERIX - Measles Schwarz Strain<br>2003: Introduction of the GSK PRIORIX vaccine
Patients vaccinated in 2003	2003: Introduction of the GSK PRIORIX vaccine - attenuated Schwarz Measles
Patients vaccinated in 2004 – 2005	2004-2005: Administration of the MERCK MMR II

Patients vaccinated between 2006 - 2010	2006-2010 (5-year tender): GSK PRIORIX - attenuated Schwarz Measles
Patients vaccinated after 2011	Beginning from 2011 we use a Sanofi-MSD product; MMRvaxPro (Measles virus Enders' Edmonston strain, live, attenuated) for vaccination and re-vaccination of children; GSK PRIORIX is still on the market, commonly used for vaccination in adulthood.
Epidemics:<br>1973-74: large epidemics, affecting primarily unvaccinated 6-9-years-old children [4]<br>1980-81: another significant epidemic, affecting primarily 7-10-years old children [4]<br>1988-89: epidemic with high age-specific attack rates of 17-21 years old individuals , who had been vaccinated during the first years of the vaccination program in Hungary [4]<br>2017-18: Smaller epidemics with few connected and sporadic cases, derived mainly from virus importation [7]	
According to the current protocol, children in Hungary get vaccinated age 15 months, and at the age of 11 years. Both vaccines are mandatory.	

Notes: Vaccination groups were defined by adding the number of months indicated for the first childhood vaccine to the dates of birth. For example, a person born in February 1990 was assigned to the group "Patients vaccinated between 1991- 1995", since this individual received the first measles (MMR) vaccine in May 1991 (date of birth + 15 months).Data were gathered using timely literature, WHO data [1,4–6,25–27] *and personal communication from experts of the National Center for Epidemiology (Budapest, Hungary). This table is also a part of our earlier publication* [8].

Table S3. Tissue Culture Infectious Dose ($TCID_{50}$) of the measles, mumps, and rubella components of different MMR and MMRV vaccines.

	Trivalent (MMR)					*Quadrivalent (MMRV)*	
Name	***Triviraten***	***MMR II***	***Morupar***	***Priorix***	***Trimovax***	***Priorix-Tetra***	***ProQuad***
Manufacturer	Berna	Merck	Sanofi Aventis	GlaxoSmit hKline	Sanofi Pasteur	GlaxoSmithKline	Merck
Measles strain	Edmonston-Zagreb	Enders Edmonston	Schwarz	Schwarz	Schwarz	Schwarz	Enders Edmonston
$TCID_{50}$	>1000	>1000	>1000	>1000	>1000	>1000	>1000
Mumps strain	Rubini	Jeryl Lynn	Urabe AM9	RIT 43857	Urabe AM9	Jeryl Lynn RIT 4385	Jeryl Lynn
$TCID_{50}$	>5000	>20,000	>5000	>5000	>5000	$>10^{4.4}$	$>10^{4.3}$
Rubella strain	Wistar RA 27/3	Wistar RA 27/3	Wistar RA 27/3	Wistar RA 27/3	Wistar RA 27/3	Wistar RA 27/3	Wistar RA 27/3
$TCID_{50}$	>1000	>1000	>1000	>1000	>1000	>1000	>1000

The original table is form: Shih-Bin Su et al., Current Status of Mumps Virus Infection: Epidemiology, Pathogenesis, and Vaccine, International Journal of Environmental Research and Public Health, 2020 [28]

Supplementary Table S4. Detailed sample numbers for the comparison represented in Figure 1.

Age	Actualized age: Original +18 yrs (2021-2003)	Equivocal	Negative	Total	(neg+equi)/total (%)	Seropos. ratio (%) (neg + equi = suboptimal)	Age group boundaries	Borcic's Sample numbers	Our current sample numbers - preliminary study - Osijek sera
1	19	4	15	70	27,14	72,86	age < 20	70	107
2	20	0	9	68	13,24	86,76			
3	21	1	7	59	13,56	86,44			
4	22	5	1	54	11,11	88,89			
5	23	4	7	59	18,64	81,36			
6	24	3	3	59	10,17	89,83			
7	25	4	6	49	20,41	79,59	20-30	sum= 597	163
8	26	0	3	51	5,88	94,12			
9	27	6	5	53	20,75	79,25			
10	28	0	5	46	10,87	89,13			
11	29	3	2	47	10,64	89,36			
12	30	0	10	52	19,23	80,77			
13	31	0	2	54	3,70	96,30			
14	32	3	6	63	14,29	85,71	31-38	sum= 236	164
15	33	4	5	47	19,15	80,85			
16-20	34-38	6	11	72	23,61	76,39			
21-30	39-48	5	12	76	22,37	77,63	39-48	76	169
31-40	49-58	1	9	80	12,50	87,50	49-58	80	143
41-50	59-68	0	0	74	0,00	100,00	59-68	74	134
over 51	over 69	1	0	72	1,39	98,61	age > 60	72	61
								1205 total sum	941 total sum

Original table from: Berislav Borc̆ic´ et al. Immunity to measles in the Croatian population, Inf. Dis. 2003 [12]

Table S5. Detailed sample numbers for the comparison represented in Figure 2.

**Original vaccination groups based on the Hungarian vaccination history (used for earlier publications)**	**From-to (years of age)**	**Seropos.ratio (%)**	**Hungarian sample numbers**	**Sample numbers of the preliminary study - Osijek sera**
before 1969	> 52	96,16105	1068	292
1969 -1977	years of age 44-52	87,55556	450	123
1987-1987	years of age 34-43	78,47826	460	197
1988-1990	years of age 31-33	89,78495	186	59
1991-1995	years of age 26-30	90,26846	298	83
1996-1998	years of age 23-25	87,92271	207	50
1999-2002	years of age 19-22	87,41497	294	41
2003	years of age 18	88,39779	181	10
2004-2005	years of age 16-17	90,11858	253	14
2006-2010	years of age 11-15	91,36691	278	30
2011-2015	years of age 6-10	93,03279	244	25
			1457 total sum	924 total sum

Original data from our previous articles: Böröcz et al.,Application of a fast and cost-effective 'three-in-one' MMR ELISA as a tool for surveying anti-MMR humoral immunity: the Hungarian experience Imm.and Inf., Böröcz et al., Imported infections versus herd immunity gaps ; a didactic demonstration of compartment models through the example of a minor measles outbreak in. Southeast Eur Med J.
